# A computational investigation of lift generation and power expenditure of Pratt’s roundleaf bat (*Hipposideros pratti*) in forward flight

**DOI:** 10.1371/journal.pone.0207613

**Published:** 2018-11-28

**Authors:** Peter Windes, Xiaozhou Fan, Matt Bender, Danesh K. Tafti, Rolf Müller

**Affiliations:** 1 Department of Mechanical Engineering, Virginia Tech, Blacksburg, Virginia, United States of America; 2 Shandong University Virginia Tech International Laboratory, Jinan, China; Coastal Carolina University, UNITED STATES

## Abstract

The aerodynamic mechanisms of bat flight have been studied using a numerical approach. Kinematic data acquired using a high resolution motion capture system was employed to simulate the unsteady air flow around a bat’s wings. A flapping bat wing contains many degrees of freedom, which make 3D motion tracking challenging. In order to overcome this challenge, an optical motion capture system of 21 cameras was used to reduce wing self-occlusion. Over the course of a meter-long flight, 108 discrete marker points on the bat’s wings (Pratt’s roundleaf bat, *Hipposideros pratti*) were tracked. The time evolution of the surface of each wing was computationally reconstructed in 3D space. The resulting kinematic model was interfaced with an unsteady incompressible flow solver using the immersed boundary method (IBM) and large eddy simulation (LES). Verification and validation of the flow simulation were conducted to establish accuracy. The aerodynamic forces calculated from the simulation compared well to the forces theoretically needed to sustain the observed flight trajectory. The transient flow field generated by the simulation allowed for the direct calculation of lift, drag, and power output of the bat during flight. The mean lift coefficient was found to be 3.21, and the flap cycle averaged aerodynamic power output was 1.05 W. Throughout the flap cycle, the planform area of the wings varied up to 46% between the largest and smallest values. During the upstroke, wing rotation was found to mitigate negative lift thereby improving overall flight efficiency. The high resolution motion capture and flow simulation framework presented here has the potential to facilitate the understanding of complex bat flight aerodynamics for both straight and maneuvering flight modes.

## Introduction

Recently, there has been growing interest in understanding and optimizing low Reynolds number flight in the regime, $\text{Re}\sim O\left(10^{2}\right)\;\text{to}\;O\left(10^{5}\right)$. For example, micro air vehicles (MAVs) have been proposed as sensing and monitoring platforms for agriculture, security, and other applications [1]. However, the aerodynamics of small flight vehicles is fundamentally different from large aircraft which operate in the high Reynolds number regime, Re ~ (10^6^) to (10^9^). Looking to nature, biological flapping fliers such as insects, birds, and bats can lend insight towards the design of MAVs [2,3].

One hallmark of flapping flight is that it remains effective at low Reynolds numbers, where the efficiency of fixed wing systems begin to decline. Flow separation over an airfoil at high angles of attack leads to the stall condition, resulting in a large drop in the lift to drag ratio [2]. In contrast, flapping flight allows for much more versatility in controlling the behavior of vortices in the flow [1,4,5]. This translates to improved flight performance over a range of Reynolds numbers.

Bats are unique from other biological fliers due to their highly articulated wing structure, and their ability to actively contour their wing membrane during flight [6–8]. Compared to birds and insects, bats exhibit greater changes in wing surface area during flight, and their wings have more degrees of freedom [9]. These features are evidenced by their ability to weave through dense forests while flying, as well as chase and capture insects [10].

The study of bat flight over the last several decades has evolved through several phases, each limited by the technology available to researchers at the time. Early research focused on morphological measurements derived from specimens, and documenting physiological parameters related to flight performance [6,11–13]. Additionally, video or multiple exposure still images were used to study basic wing kinematics during flight [14–21]. The measured flight kinematics allowed researchers to model aerodynamic forces using a steady-state flow approximation [14].

Between 2005 and 2010, improvement in high-speed digital videography allowed researchers to employ 3D stereoscopic motion capture systems to measure bat wing kinematics in wind tunnels [22,23]. Around the same time, particle image velocimetry (PIV) was employed to measure air flow in the wake of flying bats [9,24–29]. Estimates of lift and power output were made from measurements of the circulation and kinetic energy in the wakes [8,30]. This was a significant improvement over previous steady-state models, and allowed for the investigation of unsteady flow phenomena.

However, recent research has raised questions regarding the accuracy of wake based methods by suggesting that some of the underlying assumptions may not hold [31,32]. The frozen turbulence hypothesis—a prerequisite for application of the Kutta-Joukowski theorem, vortex ring model, and the actuator disk model—posits that vortices remain intact as they advect downstream into the wake, which is rarely the case in animal flight [31]. In their 2015 review on bat flight, Hedenström *et al*. noted that numerical simulation, when they become computationally feasible, have the potential to advance the state of the art and augment existing bat flight research [8].

Numerical analysis is adding a new dimension to the study of unsteady aerodynamic flows. Recently, significant progress has been made towards the simulation of animal flight aerodynamics—for example, dragonflies [33,34], cicadas [35,36], and hummingbirds [37,38]. One feature all these animals have in common is their relatively stiff wings in comparison to bats. Fewer degrees of freedom greatly simplifies both data collection as well as the flow simulations. In contrast, progress towards the simulation of bat flight is lacking in the literature. In 2014, Viswanath *et al*. conducted a first-of-its-kind aerodynamic simulation of the left wing of a fruit bat in climbing flight using the immersed boundary method [39]. The time-varying position of 49 discrete points on the bat wing drove the wing kinematics in the model. In 2015, Wang *et al*. conducted a numerical simulation of bat flight, however the wing kinematics were only defined by 10 discrete points. That is, the wing motion was dictated by the position of the shoulders, legs, and three points per wing. Based on estimates by Riskin *et al*., the skeleton alone within a bat wing contains over 20 degrees of freedom [23]. When membrane deformation is included, a minimum of 30–40 discrete points are required to fully capture the complexity of bat wing kinematics.

The goal of the present study is to examine the underlying aerodynamic mechanisms, which make bats effective fliers in the low Reynolds number regime. In the process, we aim to establish and validate a framework for capturing bat flights in 3D, and investigate it using fluid simulations. In order to achieve these goals, we demonstrate two novel advances in the study of bat flight—the use of multiple video cameras for high spatial resolution stereoscopic wing tracking, and the use of numerical flow simulations to allow for detailed examination of unsteady flight aerodynamics. These are some of the first high-resolution studies of bat flight, which give an unprecedented window into the physics of bat flight by direct determination of the time-dependent forces generated at the wing surface unlike experimental studies which attempt to deduce the mean lift forces generated by measurements in the wake.

### Motion capture of bat flight

There are several inherent challenges to conducting aerodynamic simulations of bat flight. Unlike insect wings which primarily flex, bat wings are comprised of a highly articulated skeleton which is spanned by an anisotropic, active wing membrane. These significant biological differences allow the bat wing to deform substantially during even the simplest of flight regimes. This has two major implications. First, the wings self-occlude during the flap stroke blocking aspects of the kinematics from observation. This can lead to voids in the final 3D kinematic data set if not properly addressed. Second, no simple parametric model can describe the surface of the bat wing, making the mathematical characterization of the wing shape non-trivial. For these two reasons, other methods which have been recently employed to motion capture wing kinematics of other biological fliers such as parrotlets [40], humming birds [33,41], dragonflies [34], and bees [42] are unsuitable for capturing bat flight.

In order to overcome the aforementioned challenges, a highly redundant camera array was constructed which greatly ameliorated the wing self-occlusion problem. Flight kinematic data was captured in a 1.2 m × 1.2 m cross section flight tunnel fitted with 21 cameras arranged in 3 rings about 40cm apart. The large camera array allowed for an expansive motion capture volume in addition to reducing the number of possible wing occlusions. As demonstrated in the present work, this system is capable of collecting wing kinematic data over an extended flight relative to other existing methods. Furthermore, the motion captured bat flights are not restricted in speed or direction as they are in wind tunnels, allowing for the study of maneuvering flights using the same setup.

### Aerodynamic simulation of bat flight

Another challenge is the reconstruction of the bat wing surface from the 3D kinematic data. The interplay between passive and active control of the bat wing membrane is currently not known. Thus, kinematic data collection methods which only track sparse features on the bat wing and reconstruct the surface using *a priori* assumptions about the behavior of the membrane risk mischaracterization of the wing shape. This challenge has been addressed in the present work by increasing the spatial resolution of the wing motion capture relative to other similar studies. Using a high marker density across the bat wings allowed for the direct measurement of all aspects of the wing kinematics—both skeletal joint motion as well as membrane stretching.

The present work uses the immersed boundary method to represent the bat wing in the fluid flow simulation. As other researchers have pointed out, this is an effective strategy for simulating flapping flight as it avoids the need for complex and computationally expensive re-meshing techniques [43]. Many bats, such as the one studied in the present work, fly at a Reynolds number of order 10^4^. This is a challenging intermediate Reynolds number flow to simulate since viscous effects are not negligible, requiring the solution of the full Navier-Stokes equations. Additionally, the wake does not remain strictly laminar. A parallel numerical computation on approximately 200 to 300 CPU cores was required to resolve the flow in one to four days depending on the grid resolution.

### Nomenclature

**Table:** 

*A*_*R*_	wing aspect ratio, b^2^/S
*b*	wing span
*C*_*L*_	lift coefficient
*c*_*m*_	mean wing chord
*f*	flapping frequency
*F*_*x*_, *F*_*y*_, *F*_*z*_	streamwise, lateral, and vertical aerodynamic force components
*h*_*a*_	plunge amplitude
*k*	reduced frequency
*M*	mass of bat
*Q*	wing loading
*Re*	Reynolds number
*S*	Wing planform area
*St*	Strouhal number
*U*_∞_	flight velocity
*β*	stroke plane angle
*φ*	stroke angle amplitude
*μ*	dynamic viscosity of air
*ρ*	air density

## Methods

### Flight data collection

**Fig 1:**
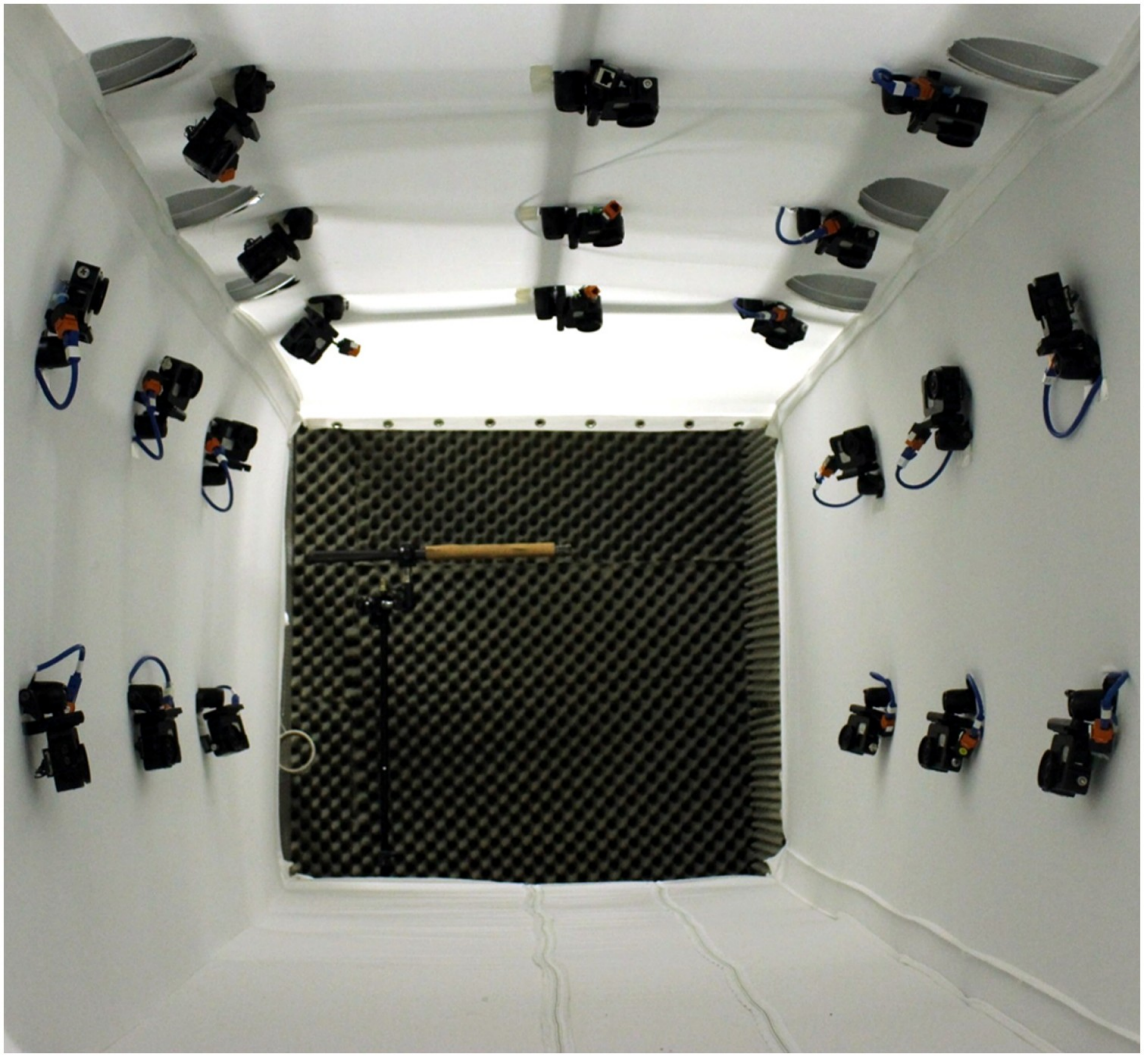
Motion capture system, consisting of 21 GoPro cameras inside a 1.2 x 1.2 meter cross section flight tunnel. The bat was trained to fly through the tunnel and land on the cork perch shown.

The motion capture system consisted of a large array of action cameras arranged inside a rectangular tunnel, as shown in Fig 1. The approximate dimensions of the tunnel were 1.2m × 1.2m × 4m. The camera model used was the GoPro Hero 3+ Black, with the resolution set to 1280×720 (720p) and the frame rate set to 120 fps. This setup allowed for the capture of up to 4 meters of bat flight. The cameras were arranged on the walls of the rectangular flight tunnel in order to observe the flying bat from various viewing directions and to minimize self-occlusion of the wings.

The cameras were synchronized using a central control unit such that all video frames were captured simultaneously (±0.1 ms) on all 21 cameras. The tunnel was illuminated using six lights mounted in the upper corners to mitigate motion blur while filming at high frame rates. The motion tracking system used in the present work is described in more detail in Bender *et al*., 2015 [44] and Bender *et al*., 2016 [45].

In the present study, bat wing kinematic data was obtained from an adult male Pratt"s roundleaf bat (*Hipposideros pratti*), an insectivorous bat indigenous to China and neighboring countries in southeast Asia. The bat was collected from a cave in southern China. Data acquisition was conducted at the Shandong University–Virginia Tech International Laboratory in Jinan, China, with oversight from Virginia Tech’s Institutional Animal Care and Use Committee (IACUC) under protocol number 15–067.

When no experiments were being conducted, the bat was kept in a small group of conspecifics in an indoor flight room (1.3 m wide, 6 m long, and 3 m high). In order to keep the bats active during the daytime experiments, the daytime and nighttime of bats were switched by lighting the bat room at nighttime for 10 hours and darkening the bat room at daytime for 14 hours. The bats were fed a daily diet of mealworms with vitamins and mineral supplements and were provided water *ad libitum*. Measurements of the particular specimen that participated in the experiments are given in Table 1.

**Table 1:** Measurements of *H*. *pratti* specimen.

Wing Span, *b*	Planform Area, *S*	Mean wing chord, *c*_*m*_	Aspect Ratio, *A*_*R*_	Mass, *M*	Wing Loading, *Q*
52.0 cm	360 cm^2^	7.4 cm	7.5	55 g	12.1 N/m^2^

The wing span is measured as the maximum tip-to-tip distance during straight flight. The planform area is the maximum area projected on the x-y plane during straight flight. The mean wing chord is calculated as *c*_*m*_ = *S*/*b*, the aspect ratio as *A*_*R*_ = *b*^2^/*S*, and the wing loading as *Q* = *M*_*g*_/*S*.

**Fig 2:**
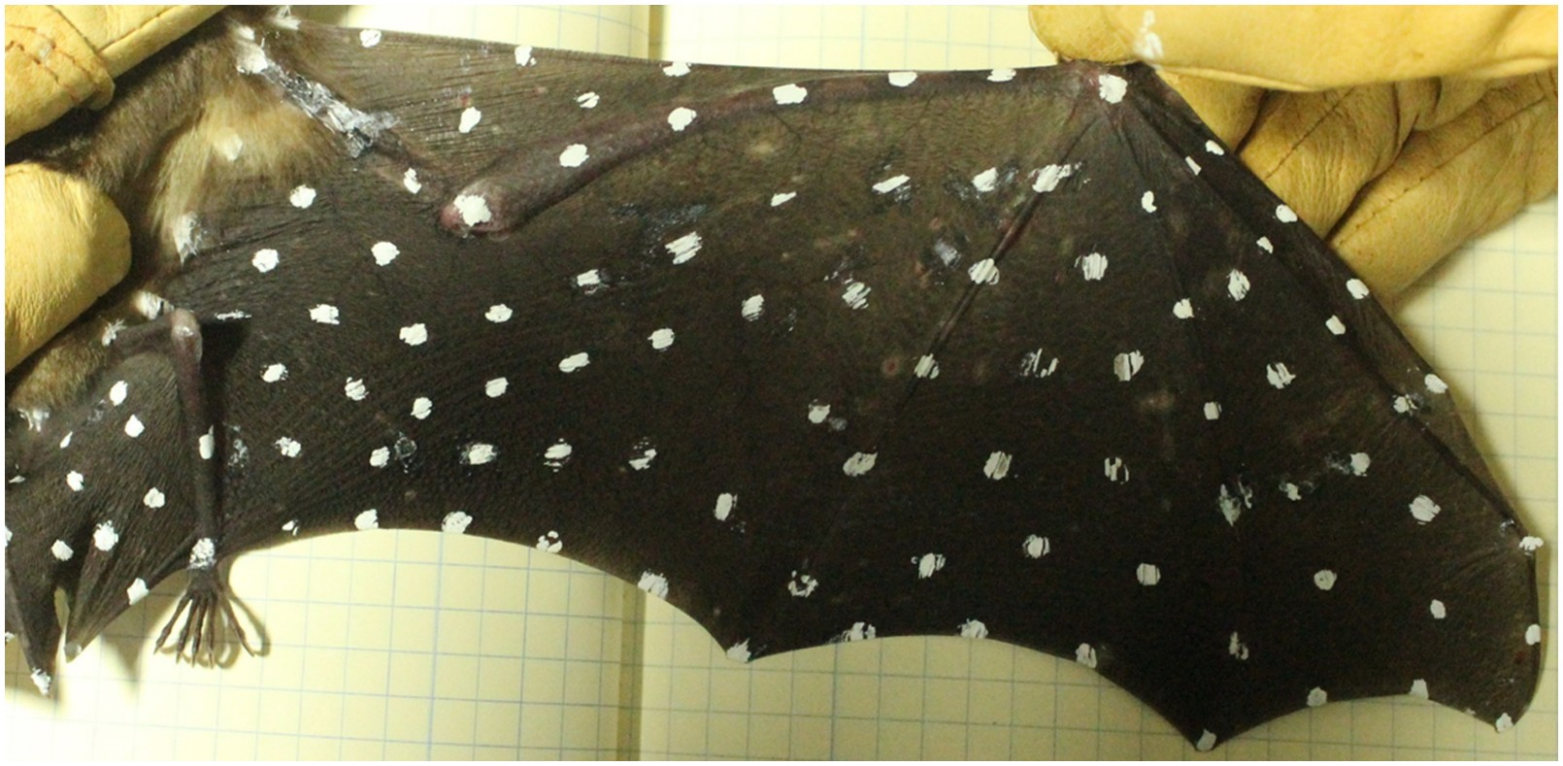
Small white markers were placed on each bat wing to aid 3D reconstruction of the wing kinematics during flight.

Multiple small white “marker points” were placed on each wing as landmarks for tracking, as shown in Fig 2. The spatial resolution of the reconstructed kinematic data was determined by how many markers were used. Using too few points to measure bat flight inadequately captures all the degrees of freedom of the bat wing. In the present work, 108 points were tracked over the course of several wing beat cycles. The final wing kinematic data set consisted of 108 discrete points at 52 instances in time.

**Fig 3:**
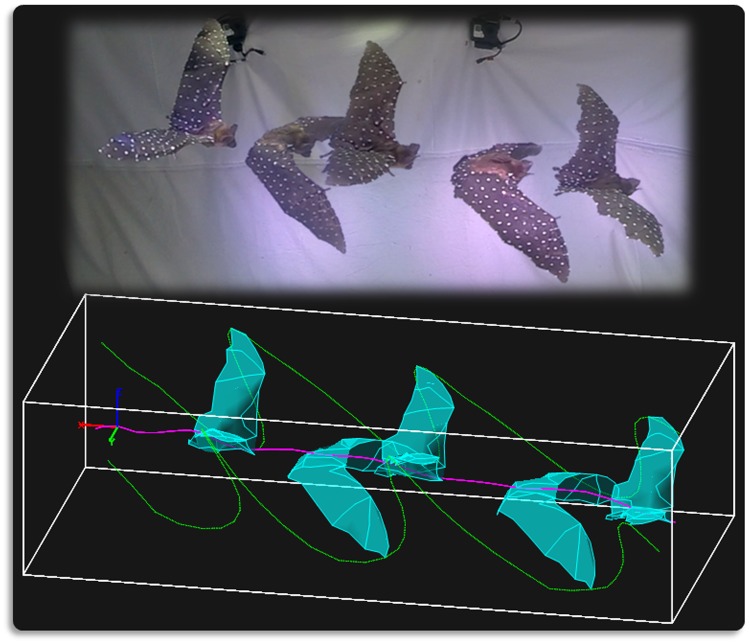
Five snapshots of the bat in flight along with the corresponding 3D reconstructed data. The radial distortion of the camera lens slightly changes the visual perspective of the bat in the video, however that is mathematically accounted for during the camera calibration process. In the digital reconstruction, the magenta trace is the path of the bat body, and the green traces are the path of the wing tips.

The cameras were calibrated using the Svoboda Multi-Camera Self-Calibration toolbox for Matlab [46]. The calibration solves for the position, orientation, and radial distortion of each of the cameras in the array such that the 3D position of any point in the scene can be reconstructed from two camera views. If more than two cameras observe the same point, multiple reconstructions of that point are generated corresponding to each camera pair. The final 3D representation was taken to be the median of the point cluster. Once the data was digitized for the entire flight, principal component analysis (PCA) was used to filter out erroneous points in a data cleaning process which is described in Fan *et al*., 2018 [47]. Finally, a time series of coordinates of each of the marker points on the bat wing was obtained. Fig 3 shows five snapshots from the flight video along with the corresponding 3D reconstruction.

### Flight simulation methodology

The aerodynamic simulations were conducted using an in-house incompressible flow solver, GenIDLEST (Generalized Incompressible Direct and Large Eddy Simulation of Turbulence) [48,49]. The flow of air over bat wings is governed by the incompressible Navier-Stokes equations,

$$\frac{\partial \vec{u}}{\partial t}+\vec{u}∙\nabla \vec{u}=-\frac{1}{\rho }\nabla p+\nabla^{2}\vec{u}$$ (1)

$$\nabla ∙\vec{u}=0$$

where $\vec{u}$ is the Cartesian velocity vector, *p* is pressure, *t* is time, *ρ* is air density, and *v* is kinematic viscosity. In GenIDLEST, solution of the Navier-Stokes equations is achieved using a finite volume framework with a second-order central difference discretization scheme for the convective and viscous terms. The pressure correction method is used with preconditioned BiCGSTAB linear solvers. Message Passing Interface (MPI) is employed for parallelization, and up to 640 CPU cores were used in the present work. Turbulence modeling was done using Large Eddy Simulation (LES). Specifically, the dynamic Smagorinsky subgrid stress model was used, where the model coefficient is calculated locally at each grid point as the simulation progresses [50,51]. The adaptive nature of the subgrid model is important due to the transitional Reynolds number of the flow (Re = 11,680).

**Fig 4:**
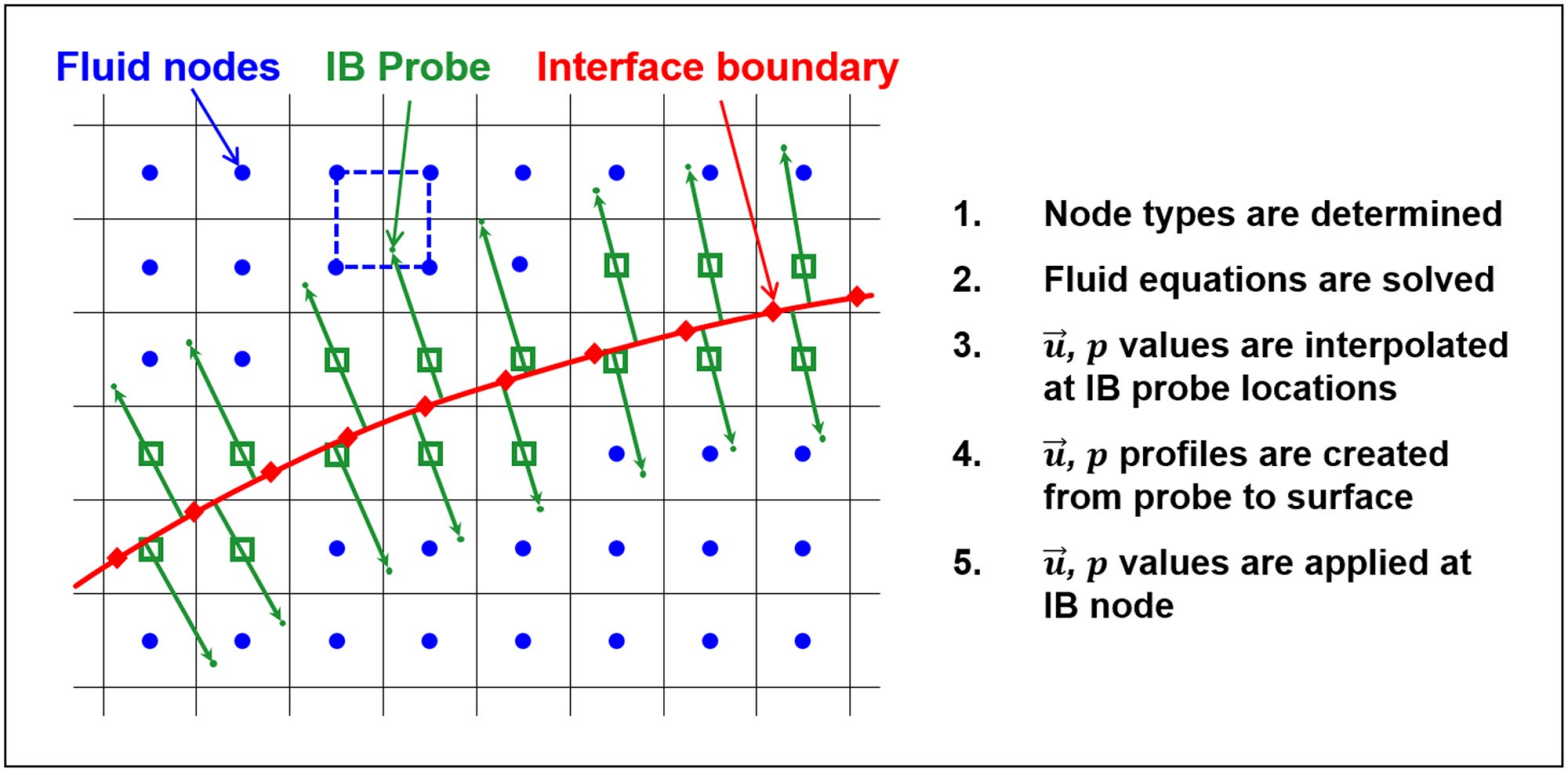
Immersed boundary method setup. A thin interface (red) is embedded into the background fluid grid. The no slip boundary condition is enforced on the interface by applying an appropriate velocity and pressure values to the IB nodes (green) at each time step. The property values at each IB node are determined by creating velocity profiles between the fluid nodes and the no slip surface.

A sharp interface immersed boundary method (IBM) was used to impose the wing surface boundary in the flow (Fig 4). The Navier-Stokes equations were solved on a structured Cartesian background grid, while the bat wing is represented by an unstructured triangular surface grid. Details of the particular implementation and validation of this IBM algorithm can be found in Nagendra *et al*., 2014 [52]. A thin surface approximation was used for the immersed surface, thus the net force on the wing was taken as the vector sum of the force on each side of a given surface element.

### Numerical implementation of flapping wings

**Fig 5:**
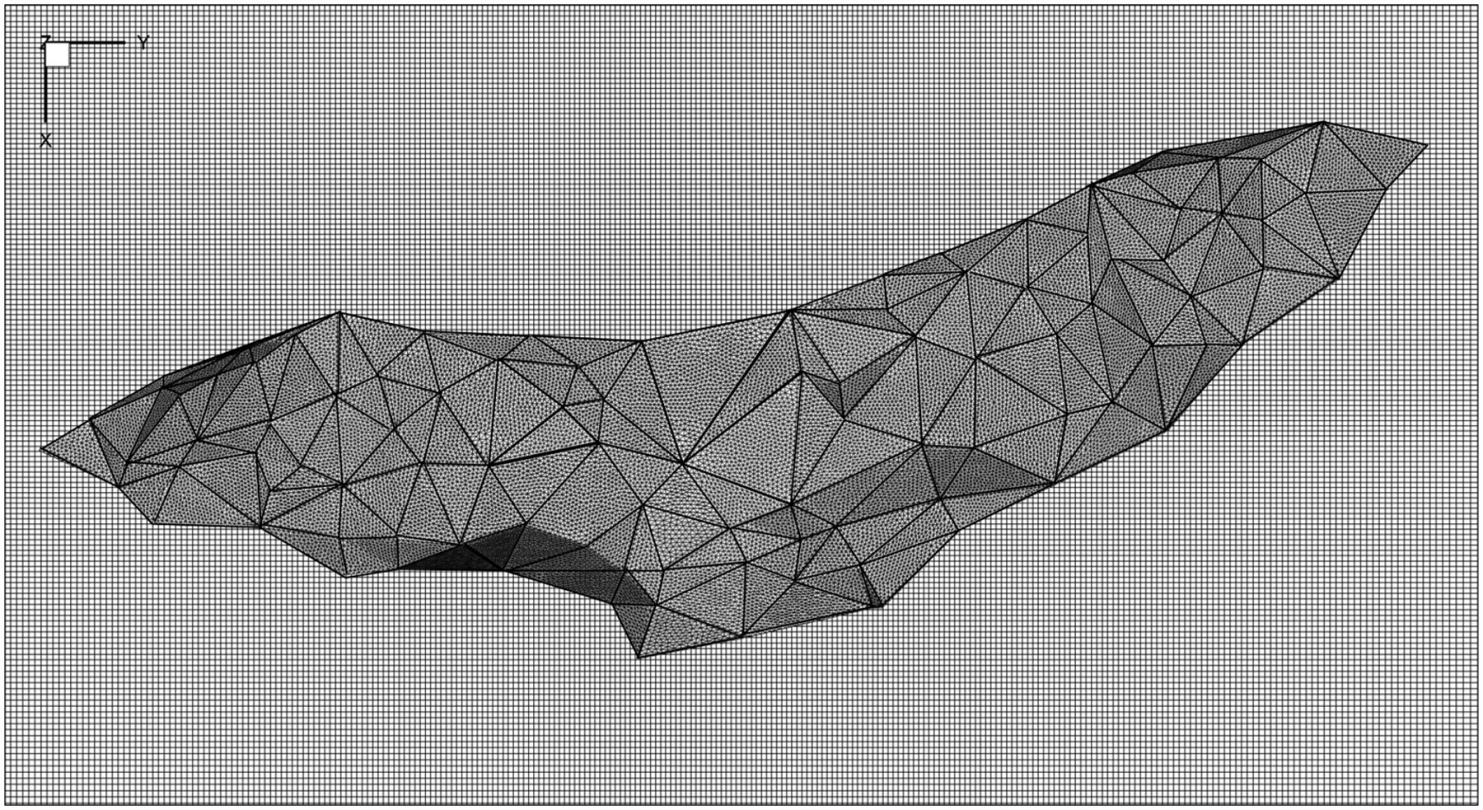
Wing surface mesh viewed from above during the most outstretched point of the downstroke. The coarse and fine wing surface meshes are showed overlaid with the fluid grid in the background. The vertices of the coarse triangular mesh correspond to the white marker points on the bat wings.

In order to represent the experimentally generated wing kinematics using the immersed boundary method (IBM), a procedure was developed to interpolate the wing surface spatially and temporally. The 3D reconstructions of the observed wing marker points are referred to in this section as “control points.” First, the control point cloud was triangulated using the Delaunay method, resulting in a coarse triangular surface mesh. This surface mesh, however, was comprised of elements with edge lengths of approximately 2 cm—too coarse for IBM simulations. Thus, the planar inner region of each control triangle was sub-meshed resulting in a finer mesh with each element edge length on the order of the background fluid mesh edge length. Finally, the wing mesh consisted of 182 control triangles, and 42,839 fine mesh elements depicted in Fig 5. The coarse mesh was used to impart the proper kinematic motion to the immersed surface, while the interpolated fine mesh was used to specify the wing surface location on the background fluid grid.

**Fig 6:**
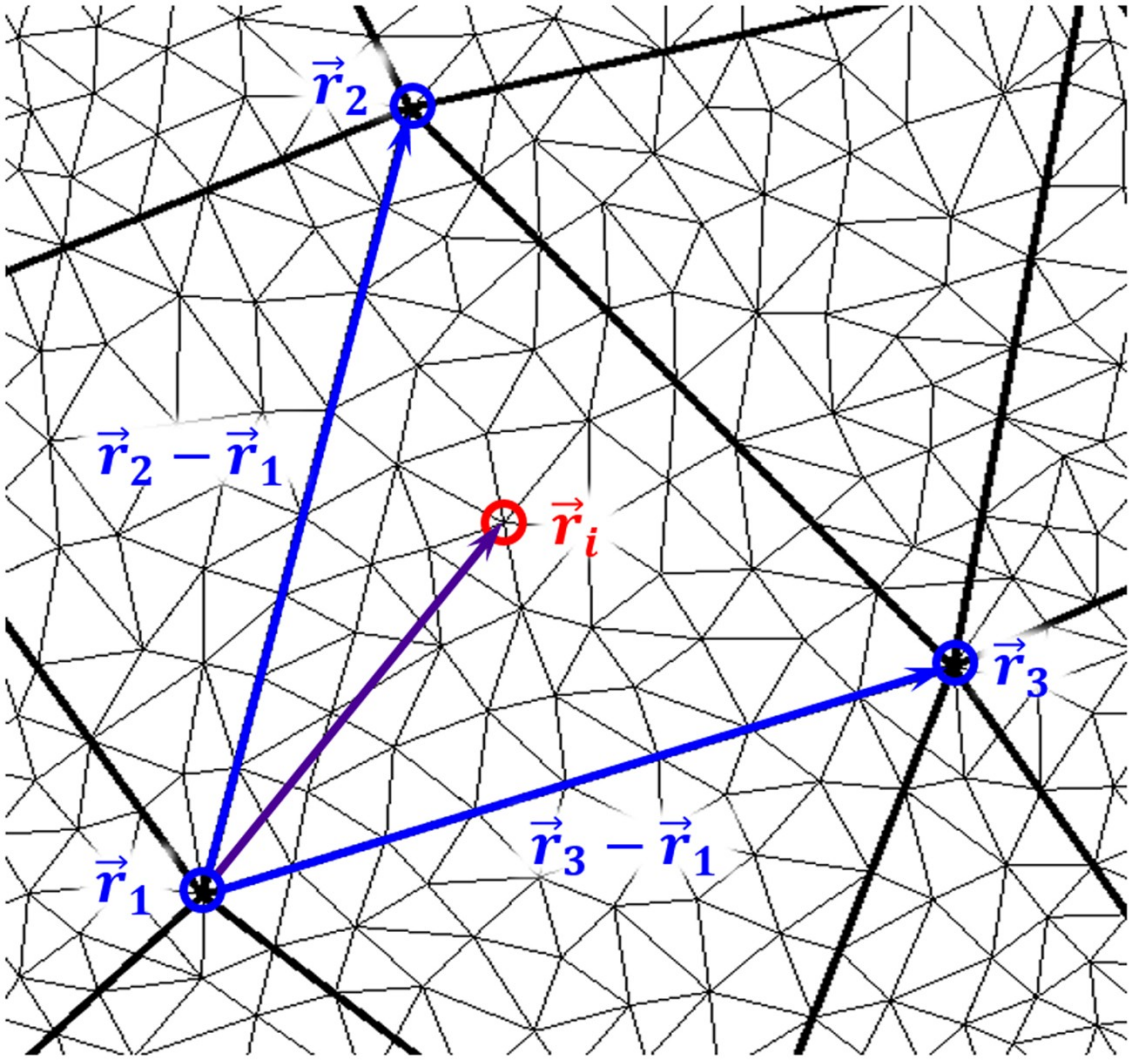
A given control triangle consists of three control points, and several interior points. The area inside the triangle is parameterized into a 2D surface, where every location is described by a unique pair of α and β values. As the wing flaps, the triangles deform and the locations of the interiors points are updated accordingly.

The location of each fine mesh vertex within a given control triangle can be described as a linear combination of two parameters—α and β—ranging between 0 and 1.

$${\vec{r}}_{i}={\vec{r}}_{1}+\alpha_{i}({\vec{r}}_{2}-{\vec{r}}_{1})+\beta_{i}({\vec{r}}_{3}-{\vec{r}}_{1})$$ (2)

where ${\vec{r}}_{i}$ is the interior point to be interpolated, and ${\vec{r}}_{1},{\vec{r}}_{2}$, and ${\vec{r}}_{3}$ are the three surrounding control point vertices, as shown in Fig 6. The fine mesh is generated based on the initial position and shape of the wings. The alpha and beta values are calculated once from the initial wing state and remain constant as the wing moves, causing the interior points to maintain their relative positions throughout the flapping cycles.

Movement of the immersed surface wing mesh is achieved using piecewise spline equations which describe the continuous evolution of each control point in time. At each time step, the spline equations are evaluated at the given simulation time. Subsequently, all fine mesh points are updated based on their respective stored α and β values as well as the updated control point locations.

Once the wing surface mesh reaches its new location at the next time instance, the centroid of each surface element is located on the computational grid using a search algorithm—that is, the element’s i,j,k computational coordinates are determined using its Cartesian x,y,z physical coordinates. This allows for proper data exchange between the fluid and the solid surface to ensure that the presence of the solid surface is manifested in the fluid field. The no-slip boundary condition is applied at the surface using the immersed boundary method. The wing surface velocity is calculated for each element using a second order backward difference scheme. Similarly, the wing acceleration is calculated using the second time derivative of the surface element locations.

### Aerodynamic simulation setup

The size of the fluid domain was set 32*c*_*m*_ × 16*c*_*m*_ × 16*c*_*m*_ along the x-, y-, and z-directions respectively. The dimensions of the cross-section of the domain approximately match the physical size of the flight tunnel. The fluid domain represents a moving reference frame that follows the bat’s flight in the x-direction at 2.5 m/s. Thus, 2.5 m/s was subtracted from the absolute x-velocity of the measured wing kinematic data. The displacement of the bat body within the fluid domain during the course of the simulation represented a perturbation relative to the moving reference frame.

**Fig 7:**
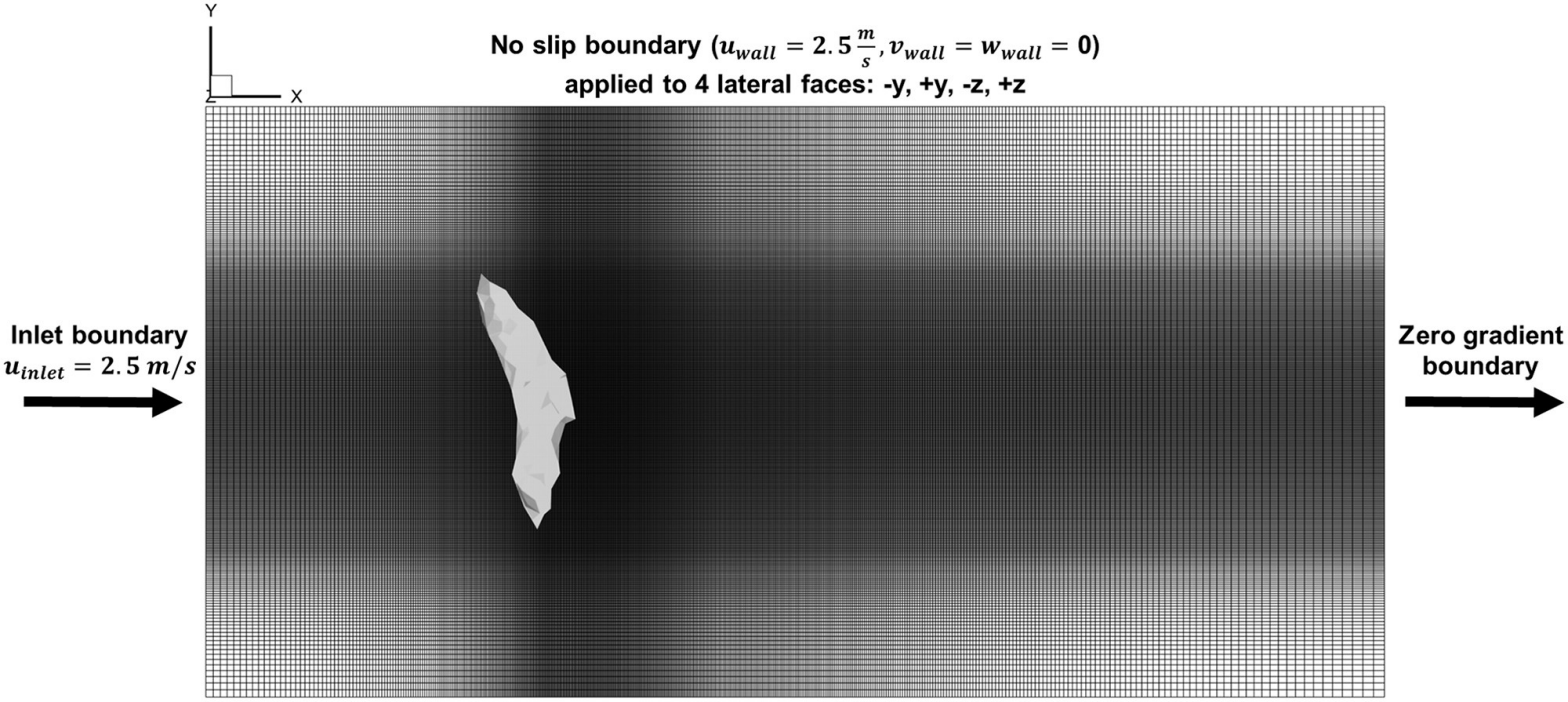
The full fluid grid viewed from above shows the refined region in the vicinity of the bat. The y- and z-faces of the domain reflect the physical size of the experimental flight tunnel, while the x-faces were positioned sufficiently far from the bat to avoid interference from the boundary.

The inlet plane and four side-wall faces of the domain were set to a constant velocity boundary condition that was equal to the reference frame velocity of 2.5 m/s. The outlet plane (+x) of the domain was set to a zero gradient boundary condition, allowing air to flow out of the domain. The bat was initially located at the origin, and the domain extends 8 chord lengths upstream of the bat and 24 chord lengths downstream of the bat as shown in Fig 7. During the course of the simulation, no fluid disturbances were observed to propagate through either the inlet or outlet boundary planes. Thus, the 32*c*_*m*_ length of the domain in the streamwise direction was deemed sufficient to prevent unphysical interference by the boundary.

The fluid domain was discretized using a non-uniform, orthogonal, Cartesian mesh for optimal computational performance. The grid contained 88.1 million fluid cells and the refined region had an average grid spacing of, Δ_*fluid*_ = 0.020 chord lengths. Thus, on average there were 50 fluid cells per chord length.

The mesh for the immersed boundary method was an unstructured triangular surface mesh which contained 21,354 vertices, and 54,238 triangular elements. The initial average edge length was Δ_*surface*_ = 0.022. As the wing flaps, the area changes and the relative position of the vertices is maintained using the movement algorithm described in Section 2.3.

### Grid independence verification

In order to validate the computational grid used in the present work, a grid independence study was conducted. Five grid resolutions were compared to determine a sufficient level of refinement. The number of fluid cells along the wing chord was used to define the grid levels. The five grids used, ranging from 12 million to 210 million fluid cells, are outlined in Table 2.

**Table 2:** Several different grids ranging from 20 fluid cell lengths per wing chord length to 100 were compared to ensure grid independence. The CPU architecture used was Intel Xeon E5-2670 (Sandy Bridge).

Cells per wing chord	Δfluid grid	Total Fluid Cells	CPU-hours	% difference |F| compared to finest
20	0.0500	11.8 million	1,350	5.9%
30	0.0333	31.5 million	2,470	3.1%
40	0.0250	62.9 million	7,130	1.9%
50	0.0200	88.1 million	7,200	1.6%
100	0.0100	209.7 million	34,330	-

**Fig 8:**
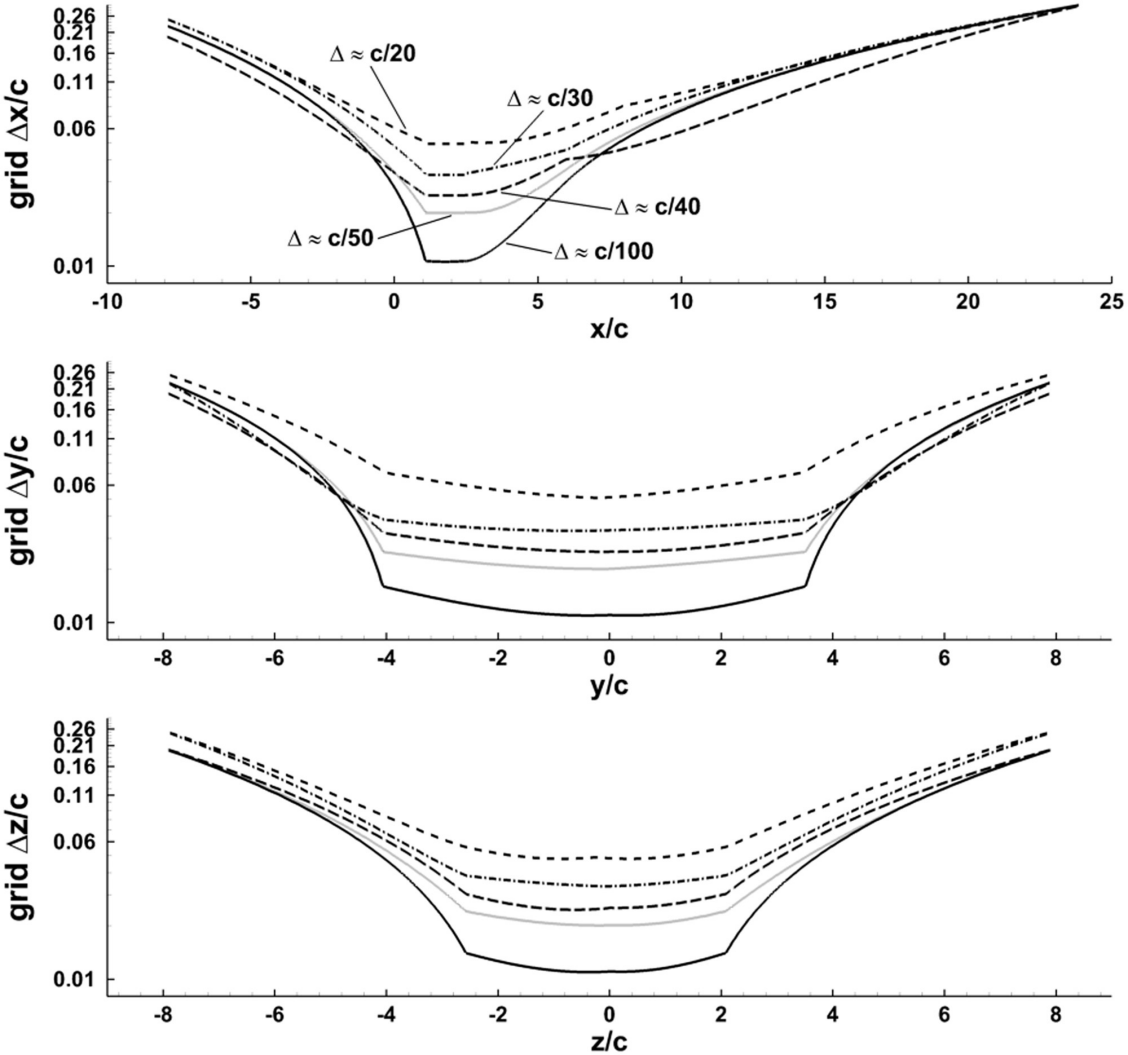
The grid spacing distribution for the five grids described in Table 2.

The fluid grid was refined in the region in which the wing surface passes, and coarsened near the edges of the domain as show in Fig 8. In doing so, the entire swept volume of the wings falls within the refined region, properly resolving the unsteady flow.

Simulations of the bat flight were run on each of the five grids. The difference in cycle averaged force magnitude between each case and the finest grid is shown in Table 2. There is a noticeable difference in force of 5.9% between the 20 and 100 cells per chord grids. However, the difference drops to 1.6% when comparing the forces from the 50 and 100 cells per chord grids which indicates close agreement between the grids. Given the uncertainties in the camera calibration and stereo triangulation, the small differences between the two finest grids can be considered negligible.

**Fig 9:**
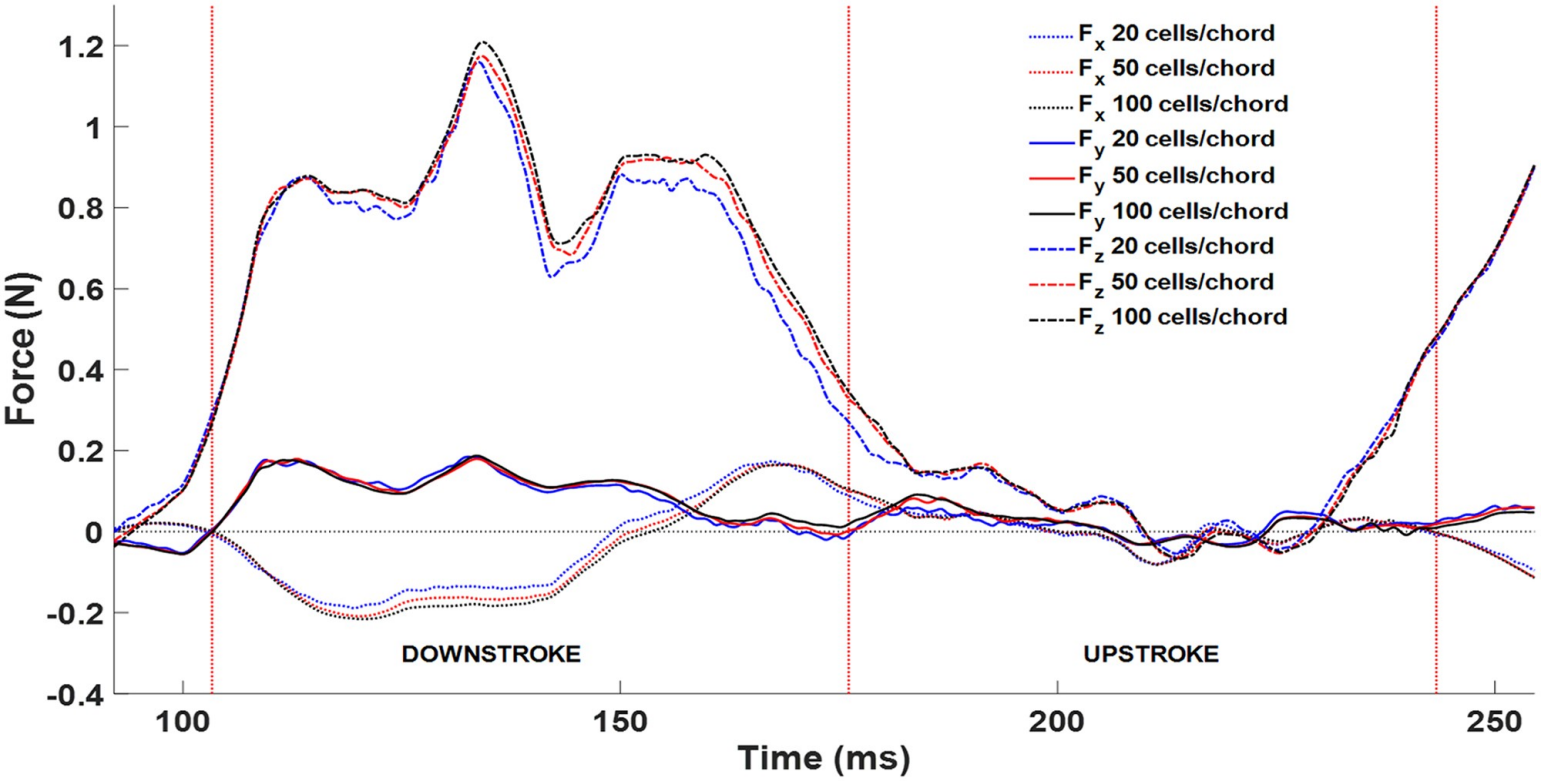
Comparison of the unsteady fluid force components for a complete flap cycle. Some discrepancy can be observed between the coarsest (blue) and finest (black) grids, however the two finest grids—50 and 100 cells per chord—show close agreement throughout the flap cycle for all three force components.

In order to further ensure grid convergence, the transient surface integrated fluid forces on the wings were compared for the 20, 50, and 100 cells per wing chord cases as shown in Fig 9. The y-dir force is close between all three grids. The x-dir force is close aside from the point of maximum thrust (-x force) between 120 ms to 160 ms. Within this time frame, the 20 cells/chord grid slightly under predicts thrust, however the 50 and 100 cells/chord grids compare closely. The z-dir force compares closely on the upstroke (180 ms to 250 ms), but differences can be observed on the downstroke around the time of peak lift production (120 ms to 180 ms). The 20 cells/chord grid consistently under predicts lift force through most of the downstroke. The 50 and 100 cells/chord grids compare well with only a few small differences, which do not materially impact the results.

## Results

### Kinematic analysis

**Fig 10:**
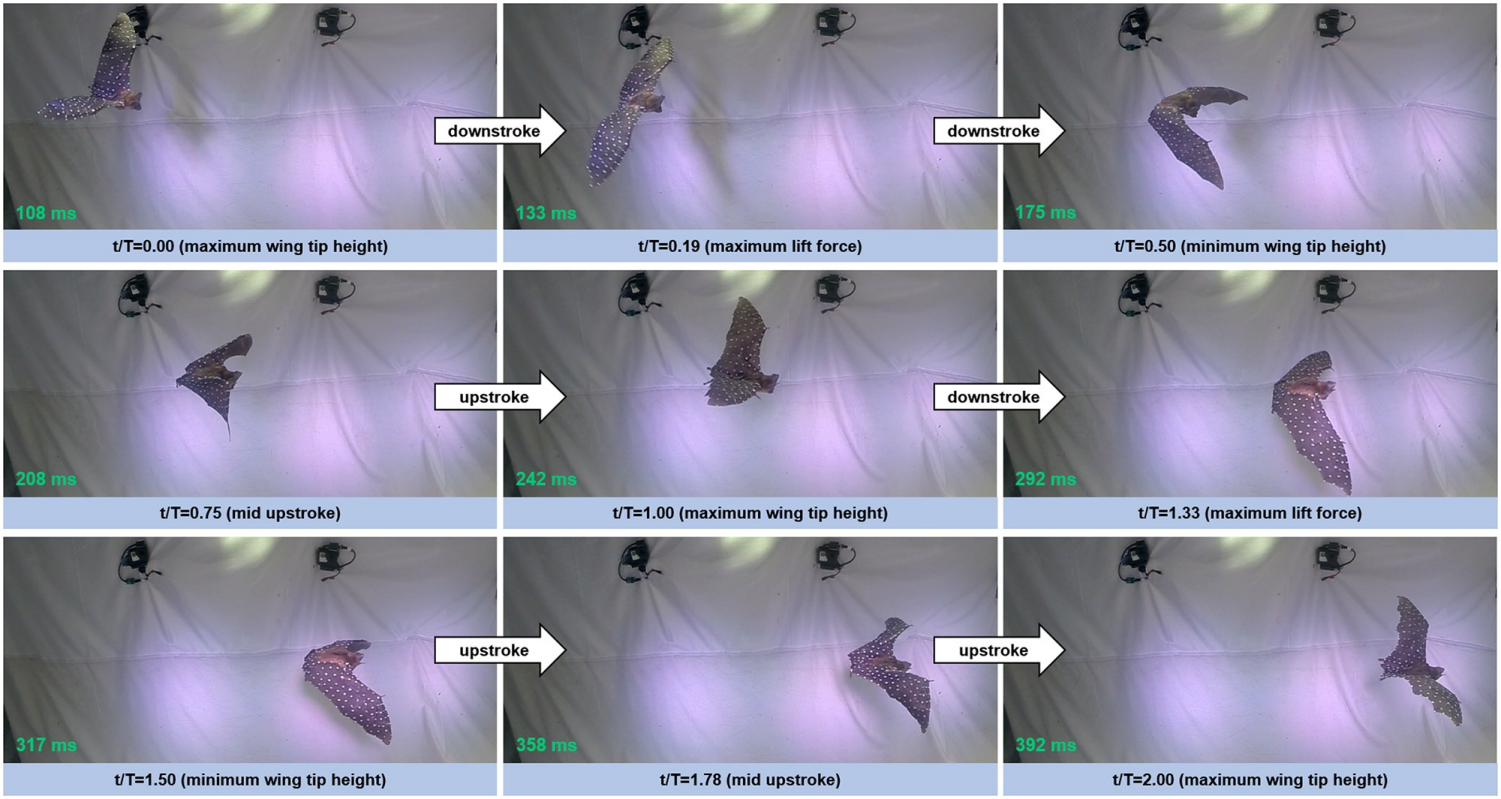
Nine frames sampled from two complete flap cycles are shown. t/T is the normalized time starting from the downstroke, and normalized by the flap period of 138 ms.

In the present study, a 1 m long flight segment was chosen for analysis. The trajectory was mostly straight and level, however the bat was slightly descending at a 5° angle and accelerating from 2.2 m/s to 2.7 m/s. This flight spanned 50 frames of video with a total duration of 0.42 s corresponding to about 3 flap cycles. Several snapshots from the flight are shown in Fig 10.

The bat had a wingspan of 52.0 cm, and an average wing chord of 7.4 cm. Thus the Reynolds number based on the mean wing chord was, Re = 11,680, and is defined as,

$$Re=\frac{\rho \;U_{\infty }\;c_{m}}{\mu }$$ (3)

where *ρ* is the air density, *U*_*∞*_ is the average flight velocity, *c*_*m*_ is the chord length, and *μ* is the air viscosity. The flow is dominated by inertia, however the Reynolds number is low enough that viscous effects cannot be neglected. The Strouhal number of the flight was, *St* = 0.61, defined as,

$$St=\frac{2\;f\;h_{a}}{U_{\infty }}$$ (4)

where f is the flapping frequency, 7.25 Hz, h_a_ is the stroke or plunge amplitude, 10.9 cm, and *U*_*∞*_ is the average flight velocity, 2.57 m/s. All the flight parameters are summarized in Table 3.

**Table 3:** Flight parameters from a 1 meter long straight flight by an *H*. *pratti*.

Average Velocity, *U*_*∞*_	Flap Frequency, *f*	Stroke or Plunge Amplitude, *h*_*a*_	Stroke Plane Angle, *β*
2.57 m/s	7.25 Hz	±10.9 cm	54.1°

The plunge amplitude is defined by half the maximum minus minimum wing tip z-positions. This value varies between the left and right wing, and between flaps so the mean was taken. The stroke plane angle is defined by the angle of the stroke plane with respect to horizontal, and describes the balance between lift and thrust. Hovering flight requires low stroke plane angles which correspond to nearly horizontal stroke planes. Conversely, accelerating flight requires a high stroke plane angle to achieve greater thrust. The value of 54.1 degrees for the present straight and level flight is typical of bats in this flight regime [53].

**Fig 11:**
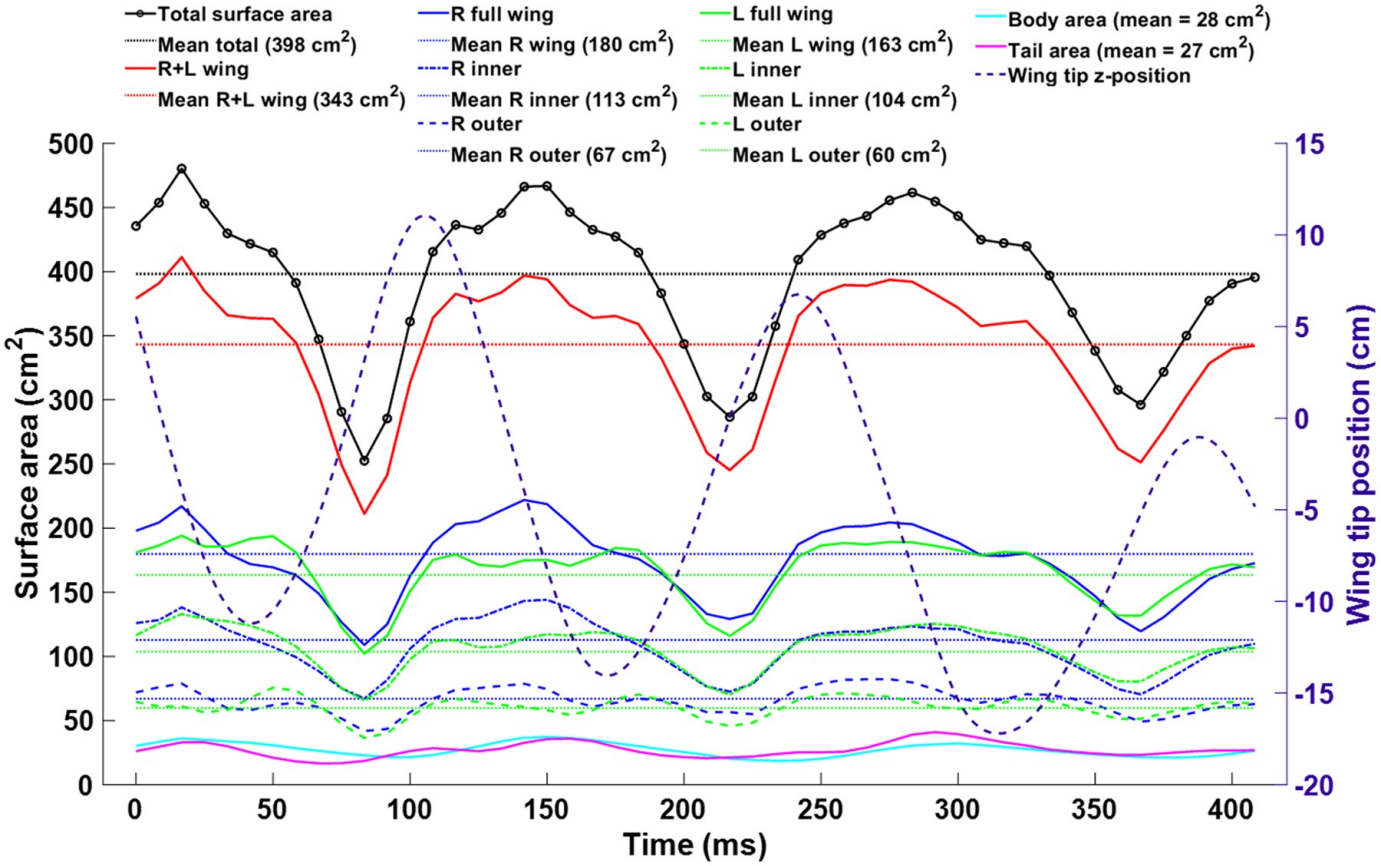
The total surface area variation of the bat wings, tail, and body is shown over the course of the flight (solid black). For context, the wing tip positions are shown (dotted black) to indicate the upstroke and downstroke.

Variations in wing surface area during flight is another aspect of the wing kinematics which can only be studied using a sufficiently spatial resolution of the wings. Fig 11 shows the time evolution of the total wing surface area along with a breakdown between different regions of the bat. The mean total surface was 398 cm^2^, and the variation was +17% and -35% for the minimum and maximum, respectively. The minimum total surface area was observed during the latter third of the upstroke, while the maximum was observed near the middle of each downstroke. The wing area begins to increase during the second half of the upstroke. This is possibly due to either active muscular control of the wing, higher air pressure under the wing as it rotates at the top of the upstroke thereby catching the flow, or a combination of both factors.

The trend observed in the wing surface area change is consistent for the left and right wing, as well as the inner and outer portions of each wing. Thus, no section of the wing was observed to experience vastly different percent change in area compared to the wing as a whole.

### Validation using lumped mass dynamics

In order to verify the flight simulation, the fluid forces exerted on the bat were examined in relation to the observed flight trajectory. The transient force output from the flow simulation was used to predict the flight trajectory of an equivalent point mass. The bat’s body mass of 55g was approximated as being concentrated between the shoulder points at the same location that the net force was applied. This allowed for a comparison that would highlight the accuracy of the numerical solution.

The transient force curve (Fig 9) was calculated by integrating the pressure and shear force over the surface of the wing at each time step. As expected, the dominate force component was lift countering the weight of the bat. For a perfectly straight and level flight, the streamwise and lateral components should be zero. Absent acceleration, the thrust and drag should exactly balance for a net zero x-force. For the present flight, the net x and y forces were indeed near zero as seen in Fig 9.

The lift, thrust, drag, lateral, and weight forces were applied to the bat. The expected acceleration of the bat due to the fluid forces at each time step was calculated by dividing the net fluid force by the mass of the bat.

$$\vec{F}(t)=m\frac{d^{2}\vec{x}(t)}{{dt}^{2}}$$ (5)

$$\vec{x}(t)=\int_{t_{0}}^{t}\int_{t_{0}}^{t}\frac{\vec{F}(t)}{m}dt\;dt$$ (6)

**Fig 12:**
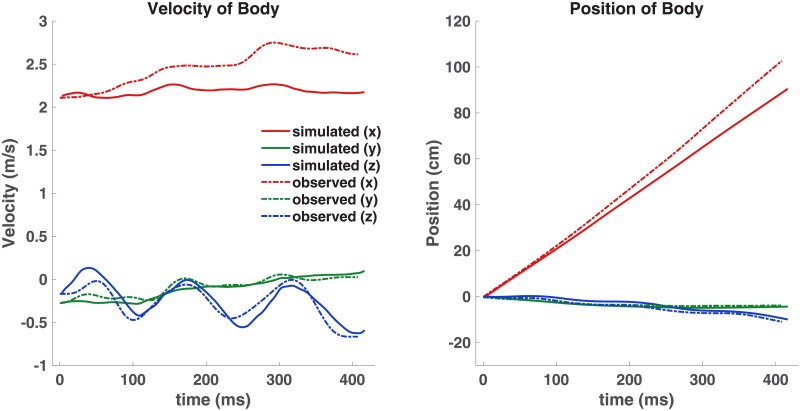
Left: Comparison of the simulated and observed velocity in x, y, and z. Right: The simulated flight trajectory is compared to the observed flight trajectory. The vertical and lateral predictions are very close, however the streamwise position is under predicted by 15 cm.

A Runge-Kutta numerical integration was performed on Eq (6) to predict the flight trajectory based on the transient lift and propulsion curves generated by the flow simulation. The trajectory, $\vec{x}(t)$, predicted from the fluid forces was then compared to the observed flight trajectory of the bat as shown in Fig 12.

The dynamics analysis approximates the mass of the bat to be a single rigid body, and ignores the wing inertia. For the assumptions made, the predicted trajectory is remarkably close to the observed flight path of the bat. The one notable discrepancy is an under prediction of the thrust force. Minimal acceleration is predicted, yet the bat accelerated from 2.2 to 2.7 m/s. The grid independence study indicated good convergence of the numerical simulation, thus the slight under-prediction of the x-location is more likely due to small errors in the wing kinematic data. On the other hand, the predicted y- and z- trajectories are very close to the observed values. Overall the dynamics analysis indicates that the forces calculated by the flow simulation are close to the actual fluid forces generated by the bat.

### Force analysis

**Fig 13:**
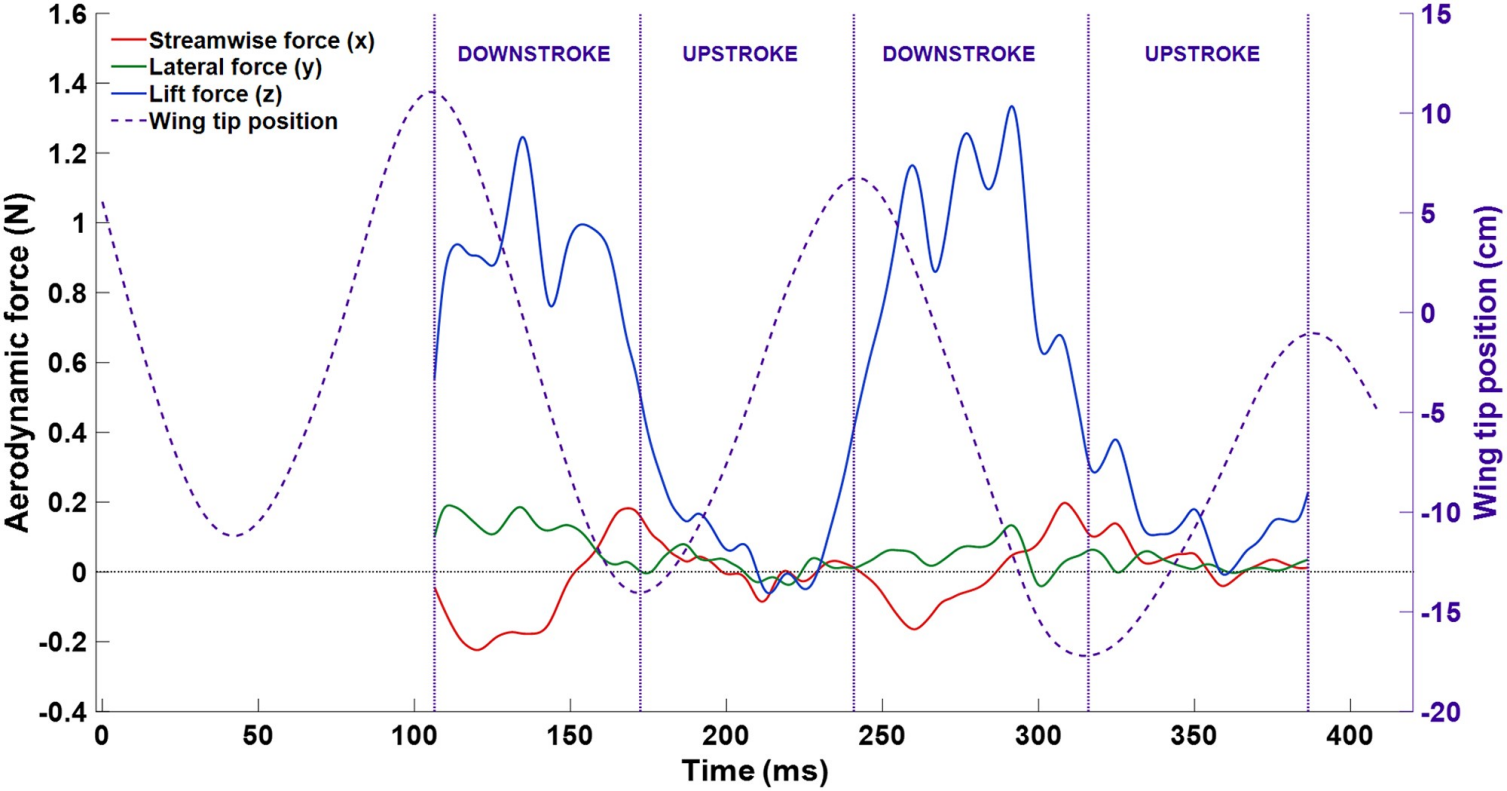
The time variation of aerodynamic force is shown along with the wing tip position for context. The peak lift force was around 1.2 N, and the cycle averaged mean was 0.525 N. The streamwise and lateral forces were both close to zero since the flight was approximately straight and level.

During flight, forces are manifested as a pressure differential across the wings. At the Reynolds number, Re ~ O(10^4^), shear stress at the wing is relatively minor compared to the pressure force, however both are included in the present analysis. The total aerodynamic force on the wings at each time instant is the surface integral,

$$\vec{F}=\int_{S}^{}(\vec{\vec{\tau }}-p\hat{n})\;dS$$ (7)

where *p* is fluid pressure, $\hat{n}$ is the wing surface normal, and $\vec{\vec{\tau }}$ is the viscous stress tensor. The resulting force curve is shown in Fig 13.

**Fig 14:**
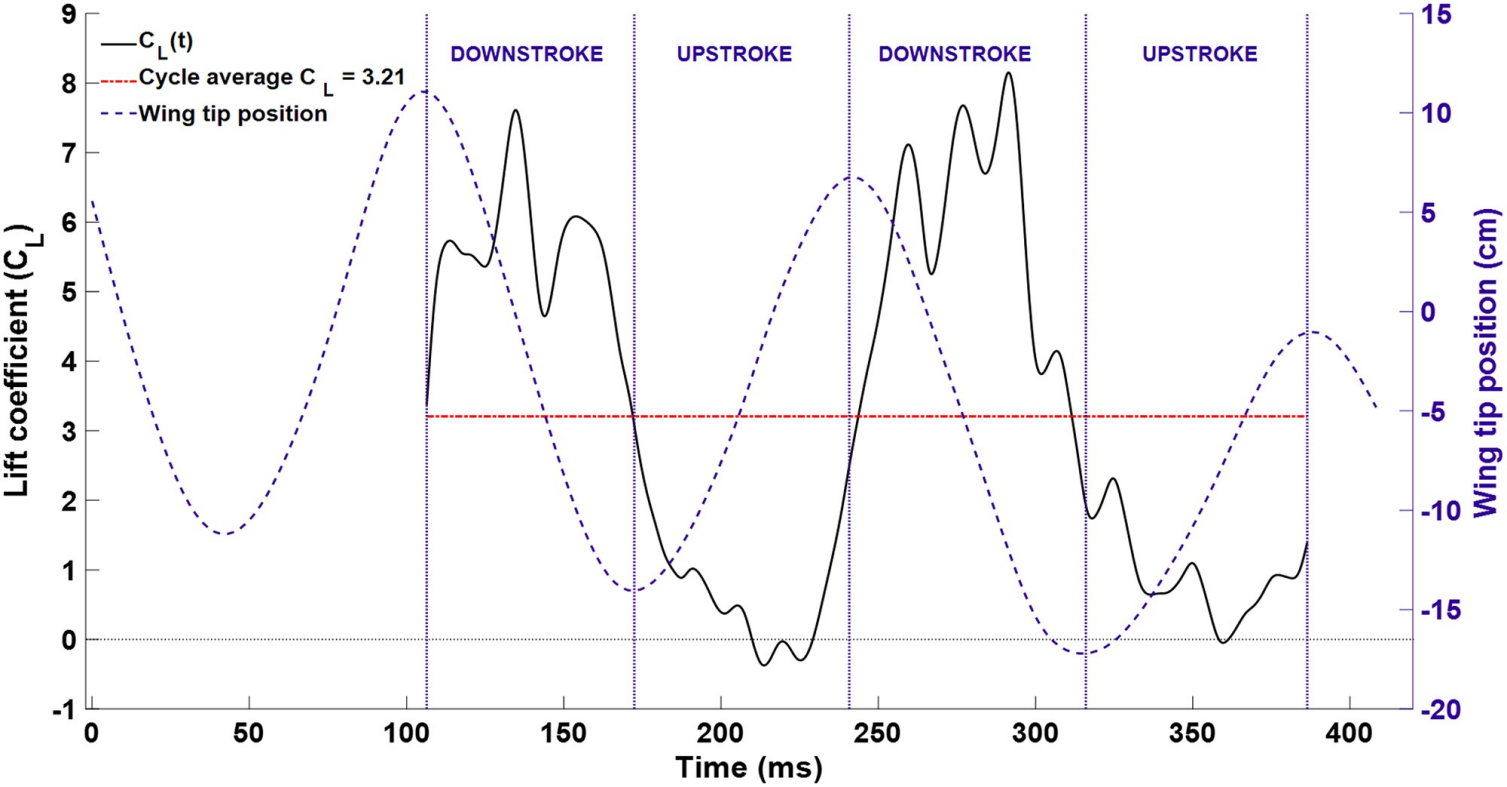
The lift coefficient is plotted along with the wing tip location for context. The mean value was *C*_*L*_ = 3.21.

The cycle averaged force values are ${\vec{F}}_{x}=0.005\;N$, ${\vec{F}}_{y}=-0.050\;N$, and ${\vec{F}}_{z}=0.525\;N$. Due to the low accelerations experienced during a mostly straight and level flight, the streamwise and lateral forces are expectedly near zero. The much larger z-component represents the lift necessary for flight. The lift can be normalized by standard methods to obtain the lift coefficient,

$$C_{L}=\frac{F_{z}}{\frac{1}{2}\rho {U_{\infty }}^{2}S}$$ (8)

where *F*_*z*_ is the dimensional vertical force, *ρ* is the air density, *U*_*∞*_ is the freestream velocity, and *S* is the maximum observed planform area of the wing during the flap cycle. The resulting values are plotted over two flap cycles in Fig 14.

The mean value of the lift coefficient was calculated to be 3.21. For fixed wing aircraft, the lift coefficient is only a function of airfoil shape and angle of attack. For this reason, it provides a convenient method to calculate lift at a given angle of attack, or calculate the proper angle of attack given a velocity. However, for bat flight, the wing shape, wing area, angle of attack, and instantaneous velocity vary continuously throughout the stroke. Others have noted the limitations of quasi-steady theory in the study of bat flight as well [8].

**Fig 15:**
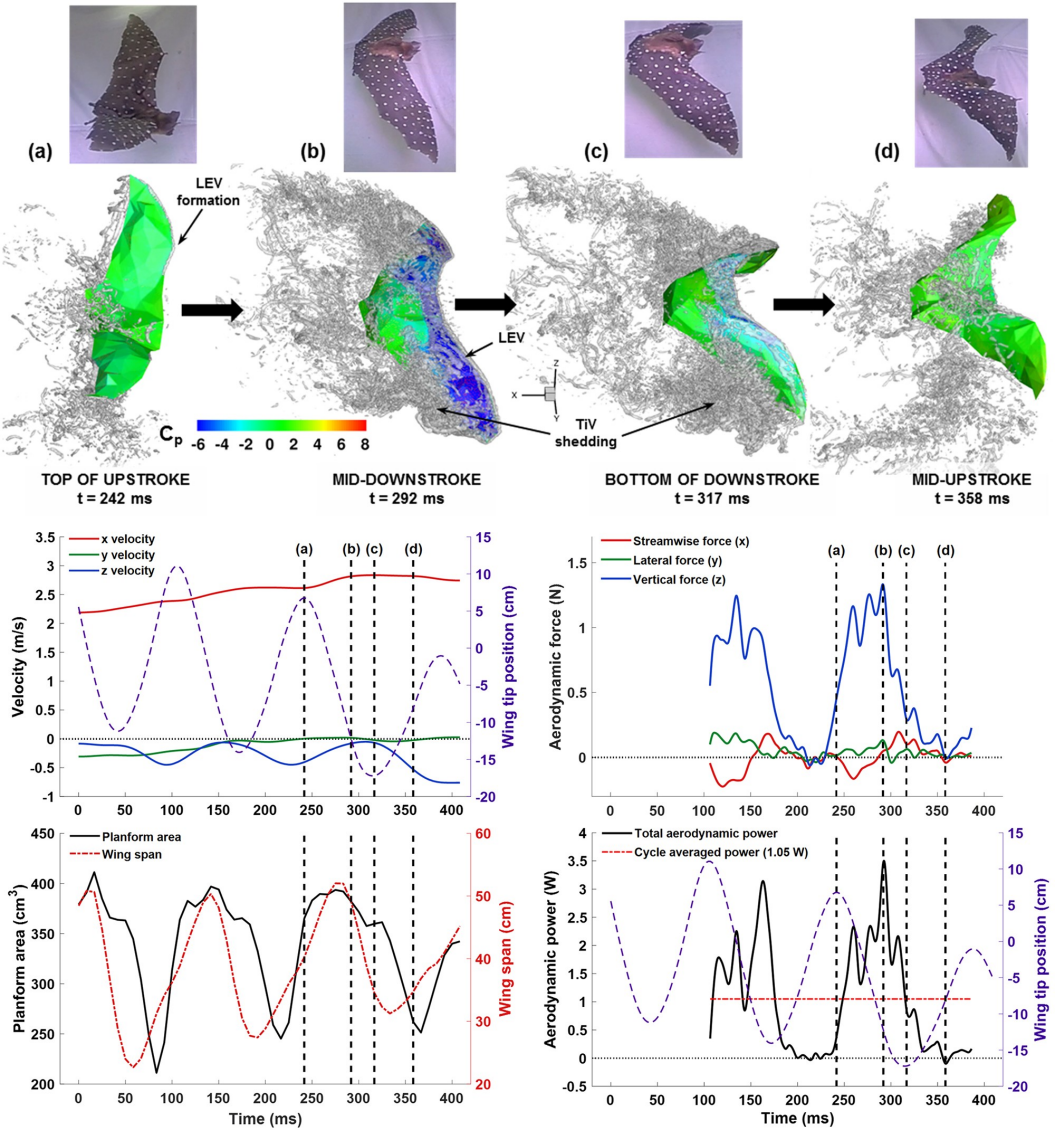
**Top: Coherent vorticity (iso-surfaces of Δ-criterion) is shown along with the wing surface pressure at four snapshots throughout the flap cycle. Bottom: Flight velocity, planform area, wing span, aerodynamic force, and aerodynamic power are plotted with each of the four snapshot locations indicated**. (a) the top of the upstroke, (b) the point of maximum lift production, (c) the bottom of the downstroke, and (d) the midpoint of the upstroke.

To understand the aerodynamic phenomena in flapping flight the unsteady flow features in relation to force production are examined. This has been the focus of recent research reviewed by Shyy et al. in 2010 [1]. Coherent vorticity is shown via Δ-criterion isosurfaces in Fig 15. Four instances in time are shown along with the corresponding flight velocity, planform area, instantaneous span, aerodynamic force, and aerodynamic power.

**Fig 16:**
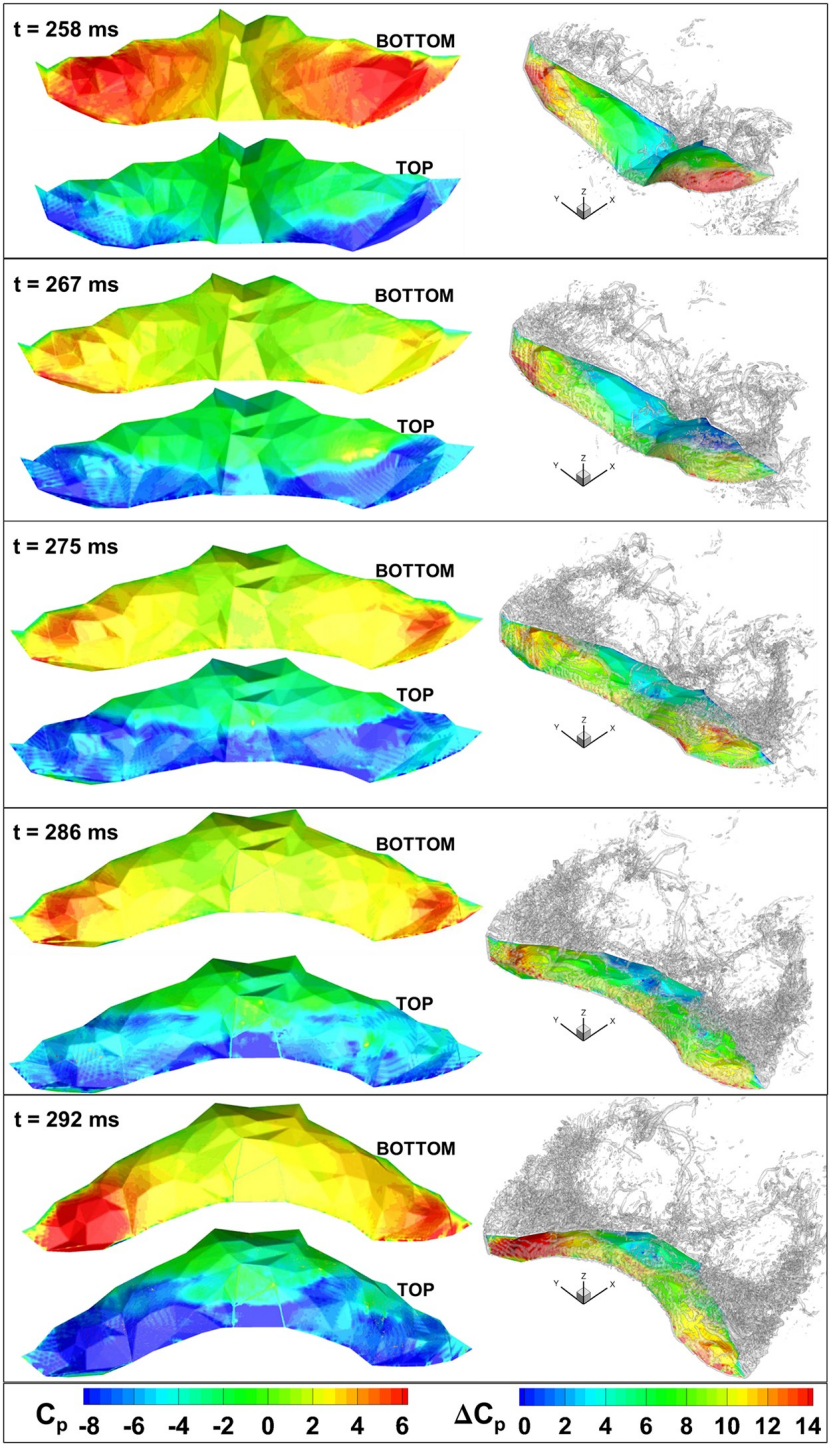
Left: Aerodynamic pressure on the top and bottom of the bat wing is shown at 5 instances during the downstroke. Right: Pressure difference is shown on the wing surface with iso-surfaces of coherent vorticity.

In Fig 15, snapshot (a) corresponds to the top of the upstroke or also the beginning of the downstroke. The lift force at (a) is about 0.45 N, or 35% of maximum lift, despite the wing having no downward velocity. Wing rotation towards the end of the upstroke as the wing readies itself for the downstroke results in a leading edge vortex (LEV) to begin to form. The aerodynamic power at time (a), as shown in Fig 15, is quite low at about 0.3 W, which is less than 10% of the maximum power expenditure. This is due to relatively low instantaneous wing flap velocity at the top of the upstroke. The planform area at time (a) has already increased to a near maximum indicating that either the freestream flow is impacting the underside of the wing stretching the membrane, or that the bat is actively stretching its wing with muscular activation. At time (a), the tip vortex (TiV) shedding is observed to be minimal. Between snapshots (a) and (b), the downward velocity of the wings accelerates rapidly. The majority of lift generation is in a 34 ms time interval from 258 ms to 292 ms which is highlighted in detail in Fig 16.

Fig 16 shows the coefficient of pressure on the bottom and top surfaces of the wing. In addition, the pressure difference—defined as the pressure under the wing minus the pressure on the top of the wing—is shown along with iso-surfaces of coherent vorticity. During both downstrokes, a notable pattern of three local maxima in the lift force can be observed (110–160 ms and 260–290 ms). Since this pattern emerges in different flap cycles, it is more likely a feature of the aerodynamics rather than random fluctuations in force. In order to explore the phenomenon in more detail, each of the five time instances shown in Fig 16 correspond to a local maxima or local minima in the force curve.

The first local maximum depicted in Fig 16, t = 258 ms, shows a coherent low pressure band on the leading edge of the wing. Moving along the chord from the leading to trailing edge, there is a distinct location, about a third of the way down the wing chord, at which the pressure rises, representing the bound of the influence of the LEV. At this instant in time, the stagnation pressure under the wing is maximum causing the large lift force—approximately 81% of the absolute maximum lift. The first local minimum in lift, t = 267 ms (Fig 16), shows the LEV convecting down the wing and breaking up slightly. Comparing to t = 258 ms, the low pressure band on the leading edge is not as complete. The stagnation pressure is also not as high at t = 267 ms compared to the previous local maximum at t = 258 ms. As such, the lift force is only 62% of the absolute maximum. At the second local maximum of lift (t = 275 ms in Fig 16), the low pressure region on the top of the wing grows in area as the LEV continues to convect along the wing. This in combination with the stagnation pressure rising slightly from t = 267 ms to t = 275 ms, causes the lift to rise to 90% of the absolute maximum. At t = 286 ms, the LEV becomes somewhat less coherent on the top surface and the low pressure region decreases in strength causing another local minimum if lift at 79% of the peak lift value.

Finally, the fifth and last instant shown in Fig 16, t = 292 ms, corresponds to the absolute maximum of lift which is also snapshot (b) from Fig 15. At this instant, a strong LEV can be seen which helps keep the flow remain attached despite a relatively high angle of attack. The LEV also maintains a strong low pressure region on the top of the wing directly contributing to lift. Flow stagnation under the wing creates an elevated pressure, which is especially pronounced towards the outer region of each wing. Additionally, prominent TiVs are shed which has also been observed in PIV experiments of bat flight [22,24,25,29,54–57]. In contrast to fix wing high Reynolds number flight, in which TiVs are known to be detrimental, flapping fliers can use TiVs to augment lift and thrust due to interactions with the leading and trailing edge vortices [1]. The point of peak lift force also is the point of maximum power expenditure at about 3.6 watts. The vertical velocity component at this time is also rising. The cyclical nature of lift production in flapping flight means that the vertical position of the bat’s body moves slightly up and down during flight.

Spanshot (c) from Fig 15 is taken at the bottom of the downstroke. Lift production drops rapidly towards the end of the downstroke—from (b) to (c) there is a nearly 90% drop in lift. The prominent TiVs can be seen convecting downstream into the wake, yet have little impact on the flight once they are shed and are significantly distanced from the wing surface. The bat begins to rotates its wings aligning them to the flow and drawing the wing tips together. At time (c) the span is about 33 cm compared to the maximum span of 52 cm. Despite the wingtip velocity dropping to zero at the bottom of the downstroke, the power expenditure is still around 1 watt, likely due to wing inertia and elastic potential being converted to aerodynamic power as the wings stop and reverse direction. The vertical velocity of the bat body is near maximum at this point, and then begins to reverse.

Spanshot (d) from Fig 15 corresponds to the mid-upstroke. The upstroke is shorter in duration than the downstroke, which allows the bat to quickly reset the wing position and prepare for the next downstroke where the majority of lift and thrust are generated. During the upstroke, the bat positions its wing perpendicular to the flow to mitigate negative lift. In fact, the lift force never drops below zero during the upstroke. The vortex structures in the flow can be seen to be minimal at this point. The planform area drops to a minimum of around 260 cm^2^ midway through the upstroke after which it starts increasing again.

### Fight power analysis

Power consumption is a particularly important metric in flight because the flight fuel must be held aloft by the flying animal or vehicle. This results in a non-linear feedback between weight and power consumption—more fuel requires more lift power which in turn requires even more fuel. Bats store fuel from food to efficiently power their wing muscles. Pennycuick [58] and others [59–63] conducted early research into the power requirements of animal flight using respiratory experiments and fixed wing airfoil theory. Other attempts to understand flapping flight power consumption involved measuring metabolic power during flight [12,64], and measuring kinetic energy in the wake of a flying animal using PIV [64].

**Fig 17:**
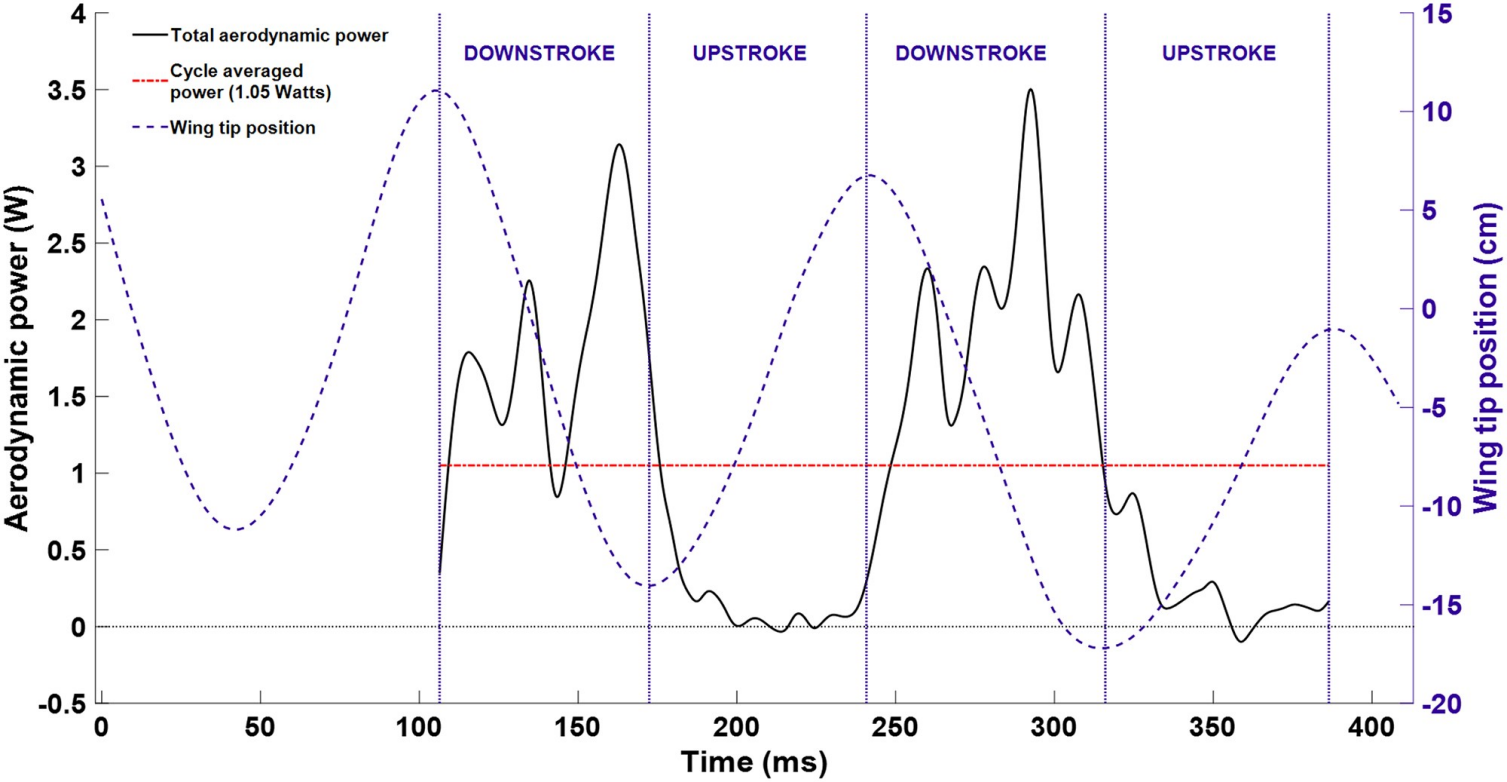
Aerodynamic power is plotted for two complete flap cycles along with wing tip position for context. Power expenditure was maximum during the second half of each downstroke at around 2.5 to 3.5 Watts. The cycle averaged value over both flaps was 1.05 Watts.

Aerodynamic simulations offer a straight forward and direct method to calculate power usage by a flying animal regardless of the complexity of the wing kinematics. At each instant in time, the aerodynamic power equals the instantaneous rate of work done by the wing on the fluid, and can be determined by,

$$P=\frac{dW}{dt}=\int_{S}^{}(p\hat{n}-\vec{\vec{\tau }})∙{\vec{v}}_{wing}\;dS$$ (9)

where *p* is fluid pressure, $\hat{n}$ is the wing surface normal, $\vec{\vec{\tau }}$ is the viscous stress tensor, and ${\vec{v}}_{wing}$ is the velocity of the wing surface. In the context of the immersed boundary method, Eq (9) is evaluated by summing $\vec{F}∙\vec{v}$ over the discrete surface elements comprising the wings. The resulting power is shown for two complete flap cycles in Fig 17.

The cycle averaged power expenditure was 1.00 W and 1.10 W for the two flaps respectively. Peak power output reached 3–3.5 W in the second half of each downstroke. In the present analysis, the wing inertia and elastic strain energy are assumed to have minimal impact on power, in line with the findings of other researchers [64]. This is largely because the wing velocity diminishes to zero at the top and bottom of the stroke, thus the inertial energy is returned to the flow. Similarly, the wing stretches during the downstroke thereby storing potential energy, but as the membrane relaxes on the upstroke the energy is returned to the flow. These factors may impact the temporal accuracy of the power values, but should not affect the cycle averaged results.

## Conclusion

Aerodynamic simulations of bat flight allow for the direct investigation of the fluid phenomena responsible for generating of lift and thrust by a flexible flapping membrane. The ability to analyze transient flow fields alongside detailed wing kinematics can advance the understanding of flapping flight beyond prior methods. Previous work in the field has leaned on the well-known theory of conventional airfoils which is not always applicable to bat flight. For example, the angle of attack, camber, and chord length vary significantly both along the span of a bat wing as well as temporally throughout the flap cycle in contrast to the well-defined and predictable behavior of a conventional airfoil. Other research has used PIV methods to measure the flow field in the wake of the bat, however this method has restrictions on the size of the bat and the ability to study flight maneuvers. Additionally, pseudo-steady wake models for force calculations rely on the frozen vortex hypothesis which does not always hold in animal flight.

Conducting aerodynamic simulations to numerically obtain the flow field around bat wings during flight is an attractive alternative to other methods of analysis. However, both high spatial resolution motion capture, as well as the numerical simulations remain a technical challenge. In the present work, 108 discrete marker points were tracked in order to fully capture the many degrees of freedom of the bat wings. After motion capture, the kinematic data was interfaced with a Navier-Stokes solver using the immersed boundary method as well as LES. A fluid grid of 88.1 million cells was used to resolve the unsteady flow of Re = 11,680.

Aerodynamic forces and the associated power were directly calculated from the resulting flow field using surface integrations. Coherent vorticity visualization was used in conjunction with wing area, force, and power data to investigate some of the underlying mechanism of bat flight. For example, while still in the upstroke, the bat starts generating lift due to wing rotation which results in the formation of a LEV. However, the majority of lift is generated during the downstroke when the LEV mitigates massive flow separation from the wing. During the upstroke, negative lift is completely avoided by virtue of wing rotation and a drastic reduction in planform area providing a net performance benefit.

There are still many challenges and open questions to be considered in the field of bat flight. For example, what is the interplay between wing elasticity and unsteady flow features? How do different bat wing morphologies manifest as optimizations to different types of flight (speed versus maneuverability versus efficiency)? What is the underlying control scheme to actuate flapping flight? How are bats so adept at maneuvering flight? Computational modeling can be a critical tool in uncovering answers to these and other open questions in the realm of bat flight. The 3D motion capture and computational framework described and validated in this study has the potential to analyze a wide variety of bat flights, including the study of maneuvering bat flight.

## Supporting information

S1 CSV File Time series of force and power data from the bat flight simulation. (CSV)

S1 Video Frames Series of images from the bat flight video. Images are provided from the view of two separate cameras. Each successive frame is separated by 1/120 seconds. The cameras were synchronized in their recording (i.e. frame 380 represents the same moment for both cameras).
(RAR)
